# Female infants are more susceptible to the effects of maternal antenatal depression; findings from the Pelotas (Brazil) Birth Cohort Study

**DOI:** 10.1016/j.jad.2020.02.025

**Published:** 2020-04-15

**Authors:** Elena Netsi, Carolina V N Coll, Alan Stein, Mariangela Freitas Silveira, Andréa Dâmaso Bertoldi, Diego G Bassani, Fernando C Wehrmeister, Marlos Rodrigues Domingues

**Affiliations:** a Department of Psychiatry, Oxford University, Oxford, UK; b Postgraduate Program in Epidemiology, Federal University of Pelotas, Brazil; c MRC/Wits Rural Public Health and Health Transitions Research Unit (Agincourt), School of Public Health, Faculty of Health Sciences, University of the Witwatersrand, Johannesburg, South Africa; d Centre for Global Child Health, The Hospital for Sick Children, Toronto, Canada; e Department of Paediatrics, University of Toronto, Canada

**Keywords:** Antenatal depression, Severe depression, Birth weight, Small for gestational age, Birth length, Sex differences

## Abstract

**Background:**

We utilised data from the 2015 Pelotas Birth Cohort, a large prospective cohort in southern Brazil, to examine the association of moderate and severe antenatal depression with child birth outcomes and explore interactions with sociodemographic characteristics.

**Methods:**

Data was available for *n* = 3046 participants and their infants. We measured antenatal depression using the Edinburgh Postnatal Depression Scale (EPDS, ≥13 for moderate and ≥17 for severe depression). Outcome measures included gestational age, birth weight, length and head circumference, using the Intergrowth-21st standards. We controlled for known confounders including obstetric risk.

**Results:**

We did not find differences in childbirth outcomes by maternal depression status for participants with at least moderate depression, although there was an increased risk for female offspring to be small for gestational age (SGA, OR 2.33[1.37,3.97]). For severe depression (EPDS≥17) we found an increased risk for lower APGAR scores (OR 1.63[1.02,2.60]) and being SGA (OR 1.77[1.06,2.97], with an increased risk for female offspring in particular to be in lower weight centiles (−10.71 [−16.83,−4.60]), to be SGA (OR 3.74[1.89, 7.44]) and in the lower 10th centile for length (OR 2.19[1.25,3.84]).

**Limitations:**

include the use of a maternal report questionnaire to ascertain depressive symptoms.

**Conclusions:**

In this recent large longitudinal cohort in Brazil we did not find independent effects of depression on adverse birth outcomes or interactions with sociodemographic characteristics. We found an increased risk of being SGA for female offspring of women with moderate and severe depression, in line with other research suggesting females may be more susceptible to antenatal disturbances.

**Funding:**

This work was supported by the Wellcome Trust, United Kingdom (095582), the Brazilian National Research Council (CNPq) and the Coordination for the Improvement of Higher Education Personnel (CAPES). EN was supported by the UK Economic and Social Research Council GCRF Postdoctoral Fellowship (ES/P009794/1).

## Highlights

• Antenatal depression of moderate severity did not raise the risk of adverse birth outcomes.
• Severe levels of antenatal depression were associated with lower APGAR scores and being SGA.
• In this group of severe antenatal depression we found an increased risk for lower weight scores, being SGA and in the lower 10^th^ centile for length for female newborns only.
• To our knowledge this is the first paper to systematically examine the potential effects of increasing severity levels of antenatal depression on birth outcomes.

## 1 Introduction

Antenatal depression has an estimated prevalence of 11% globally (Bennett et al., 2004; Gaynes et al., 2005; O'Hara and Swain, 1996), with higher rates (18–25%) (Fisher et al., 2012; Gelaye et al., 2016; Surkan et al., 2011) in low and middle-income countries (LMIC). Antenatal depression has been associated with pregnancy complications and adverse birth outcomes (Accortt et al., 2015; Alder et al., 2007; Field, 2006; Grote et al., 2010; Lundy et al., 1999; Stein et al., 2014), including alterations in brain development of infants exposed to prenatal maternal depression (Posner et al., 2016). Recent meta-analyses of observational studies suggest an increased risk of preterm birth (PTB) (Jarde et al., 2016), low birth weight (LBW) (Jarde et al., 2016) and intra-uterine growth restriction for women who experience depression during pregnancy, with larger effect sizes reported for studies conducted in LMIC (Grote et al., 2010).

Birth outcomes that may be influenced by maternal antenatal depression, such a LBW, being small for gestational age (SGA) and PTB are known predictors of poorer long-term health; increasing the risk for delayed neurodevelopment, poor linear growth (Murray et al., 2015), obesity, diabetes, hypertension and cardiovascular disease as well as entailing loss of human capital (Lawn et al., 2014). In addition, the incidence of LBW and PTB is higher in LMIC where about 1 in 5 infants are born SGA (Lawn et al., 2014).

There are many proposed mechanisms through which maternal antenatal depression may impact on foetal and child development. These include changes to the Hypothalamic Pituitary Adrenal (HPA) axis, the role of the placenta, and the contribution of genes in the heritability of stress responses and epigenetic mechanisms (Herba et al., 2016).

Research so far has predominantly relied on data from high income countries (HIC) with data from LMIC considerably under-represented. Data, such as the ones discussed in this paper, contribute significantly to the literature, not only because of the varying levels of quality of obstetric care and mental health services, but also due to the varying levels of risk factors known to raise the risk of adverse birth outcomes. Furthermore, data from settings with different confounding structures may also assist in understanding the potential causal relationship and mechanisms in play.

A number of factors may act as effect modifiers of the association between maternal antenatal depression and child birth outcomes and have not been addressed systematically in the literature. These include the persistence and severity of depression (moderate vs. severe), socioeconomic factors (for example maternal education) (Coll et al., 2017; Patton et al., 2015; Sadovsky et al., 2016; van Heyningen et al., 2016) and child sex. These have already been shown to act as moderators of the association between postnatal depression and a wide range of child development domains (Netsi et al., 2018).

There is now accumulating evidence from both HIC and LMIC (including from this cohort, Coll et al., 2017) that the likelihood of experiencing depression during pregnancy is higher in older women, women with lower levels of education (Coll et al., 2017), women reporting food insecurity and women experiencing threatening life events (van Heyningen et al., 2016). In addition, previous history of depression is one of the strongest predictors of experiencing depression during pregnancy (Coll et al., 2017; Patton et al., 2015; van Heyningen et al., 2016). These risk factors have also been associated with suboptimal birth outcomes such as LBW and intrauterine growth restriction (Sadovsky et al., 2016). Taken together this evidence demonstrates that socioeconomic and demographic inequalities impact on both perinatal mental health and birth outcomes both of which are known to affect long-term child development.

The aim of this study was to examine (i) the association between maternal antenatal depression and birth outcomes in a new prospective, longitudinal birth cohort, the 2015 Pelotas (Brazil) cohort, (ii) the role of socioeconomic and demographic moderators and (iii) the association between more severe antenatal depressive symptoms and birth outcomes.

We hypothesised that: maternal antenatal depression would be associated with LBW and PTB (<37 weeks), with the strength of the association being greater for more severe depression; and the magnitude of the association would differ across levels of socioeconomic and demographic moderators with stronger associations in the groups with lower education and income, and older maternal age groups. We also hypothesised an increased susceptibility to the effects of maternal antenatal depression for female infants.

## 2 Methods

### 2.1 Recruitment of participants & design

The 2015 Pelotas (Brazil) Birth Cohort study is a population-based prospective cohort of all children born from mothers living in the urban area of the city of Pelotas, Southern Brazil. (Hallal et al., 2017). All women with confirmed pregnancy and estimated delivery date in the year 2015 were considered eligible and invited to take part in the study. Eligible pregnant women were recruited from antenatal care health facilities including laboratories, ultrasound clinics, public primary health care units, university clinics and private clinics. Face-to-face interviews were conducted mid pregnancy (16–22 weeks of gestation) to collect information on several maternal health pregnancy-related aspects. Between January 1^st^ and December 31^st^ 2015, maternity hospitals in the city were visited daily and all eligible live births identified. In total, 4333 eligible births were identified in the five hospitals of the city (99% of performed deliveries). Newborns were examined and mothers interviewed during their stay. Fifty-one women refused to take part in the study and 7 were lost to follow-up. Refusal rate and losses were equivalent to 1.3% of eligible births (*n* = 4275). Details of the 2015 Pelotas Birth Cohort Study are available elsewhere (Hallal et al., 2017).

During the antenatal component of the study, 73.8% (*n* = 3199) of the mothers of children enroled in the birth cohort were identified, which forms the study population for this analysis. Analysis here is restricted to singleton births for whom maternal depression was assessed during pregnancy (*n* = 3046). This is due to evidence that outcomes differ substantially between singletons and twin births and because we use the INTERGROWTH 21st standards which have been calculated on singleton pregnancies. For more details on the procedures of recruitment see (Hallal et al., 2017).

The study was conducted in accordance with the ethical principles of the Declaration of Helsinki. The study was approved by the Research Ethics Committee from the Federal University of Pelotas/Superior School of Physical Education (522.064). All participants gave written informed consent.

### 2.2 Measures

#### 2.2.1 Maternal depression

Antenatal depressive symptoms were assessed using the Edinburgh Postnatal Depression Scale (EPDS). An EPDS score of ≥13 has been previously used to indicate probable major depression in this sample (Coll et al., 2017). A score of ≥17 on the EPDS is used to indicate more severe symptoms (Murray and Cox, 1990; Netsi et al., 2018; Putnam et al., 2017). The questionnaire was collected mid pregnancy (mean 21.5 weeks). We used the EPDS Portuguese translation, which has been previously validated in the 2004 Birth Cohort Study amongst postnatal Southern Brazilian women (Santos et al., 2007).

#### 2.2.2 Child outcomes

The following child outcomes were collected at birth: birth weight (g), length (cm), head circumference (cm), APGAR scores at 1^st^ and 5^th^ minutes, and gestational age at birth. Gestational age was estimated through ultrasonography or best obstetric estimate. We used the Intergrowth-21^st^ Project standards (Villar et al., 2014) to calculate standardised and centile scores for length, weight and head circumference by sex and gestational age (using the online platform https://intergrowth21.tghn.org/) for singleton newborns. Additionally, we included measures on mode of delivery (caesarean/vaginal), whether the infant was showing any health problems (no/yes), and whether the infant was admitted to the intensive care unit (no/yes).

#### 2.2.3 Confounding variables

We controlled for the following perinatal variables: maternal age (<20 years, 20–24 years, ≥35 years old), maternal schooling (0–4 years, 5–8, 9–11, 12 or more years), family income (in quintiles), skin colour (white/other), whether mother lives with partner (yes/no), maternal parity (1^st^, 2^nd^, 3^rd^), self-reported history of depression before pregnancy (no/yes), maternal pre-pregnancy body mass index (18.5–24.9, 25.0–29.9, ≥30), smoking during pregnancy (no/yes) and alcohol consumption during pregnancy (no/yes), gestational hypertension (no/yes), gestational diabetes (no/yes), eclampsia (no/yes), and child sex (male/female). The information regarding obstetric risk (gestational hypertension, diabetes, eclampsia and high pre-pregnancy BMI) was selected a priori based on previous research (do Carmo Leal et al., 2016). A proportion of women in the sample (20%) participated in the PAMELA trial (Domingues et al., 2015) which examined whether women randomised to exercise during pregnancy had improved maternal and child outcomes including maternal postnatal depression. The trial did not show any effects on the primary outcomes, we nevertheless include exercise as potential confounder to account for women's participation in this study (achieving the recommended 150 min of exercise per week throughout pregnancy/ not achieving the recommended amount of exercise).

The birth outcomes examined in this paper have also been associated with prenatal exposure to selective serotonin reuptake inhibitors (SSRIs) (Casper et al., 2003) we therefore considered adding the use of anti-depressants during pregnancy as a potential confounder. In this sample only 2.5% of the women reported anti-depressants use during pregnancy. Due to the low variability in this potential confounder we decided not to include it in the analysis. We also considered including drug use. Women were asked once during pregnancy (58.2% of the sample was asked between 16–24 weeks and 41.8% after 24 weeks). Due to the low reporting of drug use in this sample (1.1%) we did not include this variable as a potential confounder.

### 2.3 Statistical analysis

We used descriptive statistics to summarize the outcome variables and the distribution of depression in the sample, using the EPDS as a categorical score (cut-off of ≥13 to indicate depressive symptoms of at least moderate severity). We also describe birth outcomes by sex. First, we used logistic, and linear regressions to examine the association between depression and birth outcomes. Second, we examined possible interactions between depression and socioeconomic and demographic indicators (maternal education, depressive history, maternal age, multiparity, and family income) and child sex, using separate models for each potential effect modifier. We then examined the association between antenatal depression and birth outcomes including confounders which were all entered in step 2 and any observed interactions were entered in step 3. We present both the crude and adjusted models, including the interaction terms. Where the interaction term was found to be significant we also investigated the association between antenatal depression and birth outcome separately by sex. If the interaction terms were not found to be significant in the unadjusted models we did not include them in adjusted models.

We repeated the above steps to explore associations between severe depression (EPDS≥17) and child birth outcomes. For this analysis, we explored three groups using antenatal depression as an ordinal variable: women scoring <13 on the EPDS (reference group), women scoring 13–16 (moderate depression) and women scoring ≥17 on the EPDS (severe depression), using logistic regression, allowing us to investigate (i) the potential effects of progressively higher levels of depression severity and (ii) potential dose-response effects.

## 3 Results

We examined differences in key socio-demographic characteristics between the whole Pelotas birth cohort and participants with antenatal EPDS data available (*n* = 3046, supplementary Table 1). Women for whom no EPDS data was available were more likely to be younger (<20 years of age), to not be cohabiting with a partner, to have skin colour other than white, to have an unplanned pregnancy, to be smoking during pregnancy and have consumed alcohol during pregnancy, to have fewer years of education, to be in the lower income quintile and to be multipara. There were no differences by child sex.

The prevalence of maternal antenatal depression (defined as EPDS ≥13) in this sample was 16.3% and has been previously reported elsewhere (Coll et al., 2017). Participant demographic characteristics show differences by depression status in almost all variables (Table 1). Younger women (<20 years) were more likely to have scored above threshold on the depression scale. Women not cohabiting with their partner, of ethnic background other than white, women who hadn't planned the (current) pregnancy, who smoked and consumed alcohol during pregnancy, with fewer years of schooling (0–4 years) were also more likely to score above threshold on the depression scale. They were also more likely to be in the lowest income quintile, to have a history of depression and to be multipara. There were no differences in child sex.

**Table 1:** Participant Demographic characteristics (*n* = 3046) by depression status.

	EPDS <13 (*n* = 2550)		EPDS≥13 (*n* = 496)		p	Total
	N	%(95%CI)	N	%(95%CI)		N
Age at delivery	*n* = 2549		*n* = 496			*n* = 3045
<20	316	12.4(11.2,13.7)	90	18.1(15.0,21.8)	.002	406
20–34	1843	72.3(7.05,74.0)	340	68.5(64.3,72.5)		2183
≥ 35	390	15.3(14.0,16.8)	66	13.3(10.6,16.6)		456
Living With Partner	*n* = 2550		*n* = 496			*n* = 3046
No	396	15.5(14.2,17.0)	121	24.4(20.8,28.4)	<0.001	517
Yes	2154	84.5(83.0,85.8)	375	75.6(71.6,79.2)		2529
Maternal Skin Colour	*n* = 2549		*n* = 496		<0.001	*n* = 3045
White	1936	76.0(74.3,77.6)	297	59.9(55.5,64.1)		2233
Other	613	24.0(22.4,25.7)	199	40.1(35.9,44.5)		812
Planned pregnancy	*n* = 2549		*n* = 496			*n* = 3045
Yes	1342	52.6(50.7,54.6)	196	39.5(35.3,43.9)	<0.001	1538
No	1207	47.4(45.4,49.3)	300	60.5(56.1,64.7)		1507
Smoking during pregnancy	*n* = 2548		*n* = 496		<0.001	*n* = 3044
No	2248	88.2(86.9,89.4)	365	73.6(69.5,77.3)		2613
Yes	300	11.8(10.6,13.1)	131	16.4(22.7,30.5)		431
Alcohol consumption	*n* = 2548		*n* = 495		<0.001	*n* = 3043
No	2392	93.9(92.9,94.7)	440	88.9(85.8,91.4)		2832
Yes	156	6.1(5.3,7.1)	55	11.1(8.6,14.2)		211
Partner's reaction to pregnancy	*n* = 2522		*n* = 487			*n* = 3009
Pleased	2078	82.4(80.9,83.8)	355	72.9(68.8,76.6)	<0.001	2433
Indifferent	59	2.3(1.8,3.0)	27	5.5(3.8,8.0)		86
Unpleased	40	1.6(1.2,2.2)	23	4.7(3.2,7.0)		63
Doesn't live with the partner	47	1.9(1.4,2.5)	17	3.5(2.2,5.5)		64
Other	298	11.8(10.6,13.1)	65	13.3(10.6,16.7)		363
Partner support during pregnancy (perceived by pregnant woman)	*n* = 2520		*n* = 486		<0.001	*n* = 3006
High	2308	91.6(90.4,92.6)	389	80.0(76.2,83.4)		2697
Medium	117	4.6(3.9,5.5)	48	9.9(7.5,12.9)		165
Low	30	1.2(0.8,1.7)	12	2.5(1.4,4.3)		42
No support	65	2.6(2.0,3.3)	37	7.6(5.6,10.3)		102
Maternal Schooling	*n* = 2549		*n* = 496			*n* = 3045
0–4	143	5.6(4.8,6.6)	80	16.1(13.1,19.6)	<0.001	223
5–8	509	20.0(18.5,21.6)	199	40.1(35.9,44.5)		708
9–11	956	37.5(35.6,39.4)	156	31.7(27.7,35.9)		1112
12+	941	36.9(35.1,38.8)	60	12.1(9.5,15.3)		1001
Household Income (quintiles)	*n* = 2549		*n* = 495			*n* = 3044
1 (lowest)	374	14.7(13.4,16.1)	156	31.5(27.6,35.7)	<0.001	530
2	497	19.5(18.0,21.1)	120	24.2(20.7,28.2)		617
3	536	21.0(19.5,22.7)	87	17.6(14.5,21.2)		623
4	546	21.4(19.9,23.1)	89	18.0(14.8,21.6)		635
5 (highest)	596	23.4(21.8,25.1)	43	8.7(6.5,11.5)		639
Parity	*n*= 2548		*n* = 496			*n*= 3044
1st	1399	54.9(53.0,56.8)	172	34.7(30.6,39.0)	<0.001	1571
2nd	794	31.2(29.4,33.0)	168	33.9(29.8,38.2)		962
3rd	355	13.9(27.5,35.7)	156	31.5(27.5,35.7)		511
History of depression	*n* = 2546		*n* = 496			*n* = 3042
No	2211	86.8(85.5,88.1)	307	61.9(54.5,66.1)	<0.001	2518
Yes	335	13.2(11.9,14.5)	189	38.1(33.9,42.5)		524
Mother worked during pregnancy	*n* = 2549		*n* = 496			*n* = 3045
No	1012	39.7(37.8,41.6)	280	56.5(52.0,60.8)	<0.001	1292
Yes	1537	60.3(58.4,62.2)	216	43.5(39.2,48.0)		1753
Gestational Hypertension	*n* = 2549		*n* = 496		<0.012	*n* = 3045
No	1920	75.3(736.,77.0)	347	70.0(65.8,73.8)		2267
Yes	629	24.7(23.0,26.4)	149	30.0(26.2,34.2)		778
Eclampsia	*n* = 2542		*n* = 495			*n* = 3037
No	2382	93.7,92.7(94.6)	458	92.5(89.8,94.5)	.329	2840
Yes	160	6.3(5.4,7.3)	37	7.5(5.5,10.2)		197
Gestational Diabetes	*n* = 2549		*n* = 496			*n* = 3045
No	2319	91.0(89.8,92.0)	427	86.1(82.8,88.9)	.001	2746
Yes	230	9.0(8.0,10.2)	69	13.9(11.1,17.2)		2990
Pre-pregnancy BMI≥30	*n*= 2502		*n* = 479			*n* = 2981
BMI <18.5	78	3.1(2.5,3.9)	19	4.0(2.5,6.1)	.289	97
BMI 18.5 - <25.0	1240	49.6(47.6,51.5)	225	47.0(42.5,51.5)		1465
BMI 25.0 - <30.0	740	28.1(26.4,29.9)	128	26.7(22.9,30.9)		832
BMI ≥30	480	19.2(17.7,20.8)	107	22.3(18.8,26.3)		587
Exercise during pregnancy	*n* = 2545		*n* = 496		<0.001	*n* = 3041
150 mins per week	306	12.0(10.8,13.3)	33	6.7(4.8,9.2)		339
Less than 150 mins per week	2239	88.0(86.7,89.2)	463	93.3(90.8,95.2)		2702
Sex of the child	*n* = 2550		*n* = 496		0.636	*n* = 3046
Male	1288	50.5(48.5,52.4)	256	51.6(47.2,56.0)		1544
Female	1262	49.5(47.6,51.5)	240	48.4(44.0,52.8)		1502

### 3.1 Child birth outcomes

#### 3.1.1 Moderate depression

##### 3.1.1.1 Main effects

Table 2 presents the description of child birth outcomes for the whole sample and by maternal antenatal depression status. Table 3 presents birth outcomes by child sex. Table 4 presents the unadjusted and adjusted analyses. For neonates of women who scored above the depression threshold during pregnancy there was an increased risk of being shorter in length (standardised and centile scores, coefficient −0.12 (−0.22,−0.010) and −3.37(−6.21,−0.53)), being of LBW (standardised and centile scores −0.11 (−0.021,−0.0.2) and −3.20 (−5.93,−0.47) and SGA (OR 1.48 [1.04, 2.09]). We found a difference in type of delivery with women scoring above threshold on the depression scale more likely to have caesarean delivery compared to vaginal (OR 0.74 [0.60,0.90]). We also present the models considering confounders (as outlined in the methods section). Inclusion of confounders in the models attenuated the effects reported above (Table 4).

**Table 2:** Birth outcomes by maternal antenatal depression status. Pelotas, 2015 Birth Cohort.

Variables	Antenatal Depression (*n* = 496)		EPDS <13 (*n* = 2550)		p	Total N (*n* = 3046)
	N	%(95%CI)	N	%(95%CI)		N
APGAR Scores						
APGAR 1 min	*n* = 494		*n* = 2546			*n* = 3040
High (≥7)	442	89.5(86.4,91.9)	2327	91.4(90.2,92.4)	.170	2769
Low <7	52	10.5(8.1,13.6)	219	8.6(7.6,9.8)		271
APGAR 5 min	*n* = 495		*n* = 2547			*n* = 3042
High (≥7)	492	99.4(98.1,99.8)	2518	98.9(98.4,99.2)	.288	3010
Low <7	3	0.6(0.2,1.9)	29	1.1(0.8,1.6)		32
Newborn health problems	*n* = 496		*n* = 2546			*n* = 3042
No	444	89.5(86.5,91.9)	2302	90.4(89.2,91.5)	.536	2746
Yes	52	9.6(8.5,10.8)	244	9.6(8.5,10.8)		296
Admitted to NICU	*n* = 496		*n* = 2550			*n* = 3046
No	452	91.1(88.3,93.3)	2366	92.8(91.7,93.7)	.200	2818
Yes	44	8.9(6.7,11.7)	184	7.2(6.3,8.3)		228
Length for age Z-score^1^	*n* = 493	-0.29(1.10)	*n* = 2543	-0.17(1.07)	.026	*n* = 3036
Length (cm)^1^	*n* = 493	42.20(29.24)	n = 2544	45.6(29.45)	.200	*n* = 3037
Length (centiles)	*n* = 493		*n* = 2544			*n* = 3037
<10th centile	84	83.0(79.4,86.0)	2190	86.1(84.7,87.4)	.071	438
≥10th centile	409	17.0(14.0,20.6)	354	13.9(12.6,15.3)		2599
Head circumference for age Z-score^1^	*n* = 493	0.32(1.18)	*n* = 2544	0.37(1.1)	.210	*n* = 3037
Head circumference (cm)^1^	*n* = 493	57.68(29.29)	*n* = 2546	59.79(28.49)	.135	*n* = 3039
Head circumference (centiles)	*n* = 493		*n* = 2546			*n* = 3039
<10th centile	30	93.9(91.4,95.7)	141	94.5(93.5,95.3)	.629	171
≥10th centile	463	6.1(4.3,8.6)	2405	5.5(4.7,6.5)		2868
Birth weight for age Z-score^1^	*n* = 495	0.13(1.02)	*n* = 2546	0.25(1.01)	.021	*n*=3041
Birth weight (centile)^1^	*n*= 495	53.86(28.88)	*n* = 2547	57.06(28.23)	.022	*n*=3042
Small for Gestational age (SGA)	*n* = 495		*n* = 2547			*n* = 3042
<10th centile	44	91.1(88.3,93.3)	2389	93.8(92.8,94.7)	.028	202
≥10th centile	451	8.9(6.7,11.7)	158	6.2(5.3,7.2)		2840
Large for Gestational age (LGA)	*n* = 495		*n* = 2547			*n* = 3042
>90th centile	56	11.3(8.8,14.4)	368	14.4(13.1,15.9)	.650	424
≤90th centile	439	88.7(85.6,91.2)	2179	85.6(84.1,86.9)		2618
Gestational Age at birth (weeks)^1^	*n* = 496	38.69(2.06)	*n* = 2550	38.61(1.98)	.404	*n* = 3046
<37	61	87.7(84.5,90.3)	329	87.1(85.7,88.3)	.713	390
≥ 37	435	12.3(9.7,15.5)	2221	12.9(11.7,14.3)		2656
Mode of Delivery	*n* = 496		*n* = 2549			*n* = 3045
Vaginal	195	39.3(35.1,43.7)	823	32.3(30.5,34.1)	.002	1018
Caesarean section	301	60.7(56.3,64.9)	1726	67.7(65.9,69.5)		2027

NICU: Neonatal Intensive Care Unit.

1 mean and standard deviation.

**Table 3:** Birth outcomes by child sex. Pelotas, 2015 Birth Cohort.

Variables	Males *n* = 1541		Females *n* = 1502		p
APGAR Scores	*n*	%(95%CI)	*n*	%(95%CI)	
APGAR 1 min					
High (≥7)	1393	90.6(89.0,91.9)	1375	91.7(90.2,93.00	.262
Low <7	145	9.4(8.1,11.0)	124	8.3(7.0,9.8)	
APGAR 5 min					
High (≥7)	1519	98.6(97.9,99.1)	1489	99.3(98.8,99.6)	.056
Low <7	21	1.4(0.9,2.1)	10	0.7(0.4,1.2)	
Newborn health problems					
No	1370	89.0(87.3,90.4)	1376	91.6(90.1,92.2)	.014
Yes	170	11.0(9.6,12.7)	126	8.4(7.1,9.9)	
Admitted to NICU					
No	1406	91.2(89.7,92.6)	1409	93.8(92.5,94.9)	.007
Yes	135	8.8(7.4,10.3)	93	6.2(5.1,7.5)	
Length for age Z-score^1^	1538	−0.18(1.08)	1498	−0.21(1.07)	.413
Length (cm)^1^	1538	45.89(29.5)	1499	44.14(29.3)	.101
Length (centiles)					
<10th centile	1303	84.7(82.8,86.4)	1296	86.5(84.6,88.1)	.173
≥10th centile	235	15.3(13.6,17.2)	203	13.5(11.9,15.4)	
Head circumference for age Z-score^1^	1538	0.43(1.14)	1499	0.29(1.08)	<0.001
Head circumference (cm)^1^	1539	61.13(28.47)	1500	57.71(28.7)	.001
Head circumference (centiles)					
<10th centile	81	5.3(4.3,6.5)	90	6.0(4.9,7.3)	.378
≥10th centile	1458	94.7(93.5,95.7)	1410	94.0(92.7,95.1)	
Birth weight for age Z-score^1^	1541	0.25(1.01)	1500	0.21(1.01)	.987
Birth weight (centile)^1^	1541	57.2(28.3)	1501	55.9(28.4)	.196
Small for Gestational age (SGA)					
<10th centile	96	6.2(5.1,7.6)	106	7.1(5.9,8.5)	.357
≥10th centile	1445	93.8(92.4,94.9)	1395	92.9(91.5,94.1)	
Large for Gestational age (LGA)					
>90th centile	225	14.6(12.9,16.5)	199	13.3(11.6,15.1)	.285
≤90th centile	1316	85.4(83.5,87.1)	1302	86.7(84.9,88.4)	
Gestational Age at birth (weeks)		38.61(1.96)		38.65(2.00)	.567
<37	207	13.4(11.8,15.20	181	12.1(10.5,13.8)	.253
≥ 37	1334	86.6(84.8,88.2)	1321	87.9(86.2,89.5)	
Mode of Delivery					
Vaginal	488	31.7(29.4,34.1)	530	35.3(32.9,37.7)	.036
Caesarean section	1052	68.3(65.9,70.6)	972	64.7(62.3,67.1)	

NICU:Neonatal Intensive Care Unit.

1 mean and standard deviation.

**Table 4:** Crude and adjusted analysis for the association between maternal antenatal depression of at least moderate severity and birth outcomes for outcomes with. Pelotas, 2015 Birth Cohort.

		**Logistic Regression**		
Variables	Crude OR (95%CI)	Interaction with gender in crude model	Adjusted OR (95%CI)	Interaction with gender in adjusted model
APGAR 1 min (<7)	1.25(0.91,1.72)	1.64(0.86,3.10), *p*=.131	1.16(0.8,1.66)	
APGAR 5 min (<7)	0.53(0.16,1.74)	1.17(0.09,14.75), *p*=.903	0.47(0.13,1.70)	
Newborn health problems (yes)	1.10(0.81,1.52)	0.93(0.49,1.77), *p*=.827	0.88(0.61,1.27)	
Admitted to NICU (yes)	1.25(0.89,1.77)	0.92(0.46,1.87), *p*=.824	1.09(0.73,1.64)	
Length (<10th centile)	1.27 (0.98,1.65)	**2.01(1.19,3.40), *p*=.009**	1.05(0.78,1.41)	**2.00(1.15,3.46), *p*=.014**
Head circumference (<10th centile)	1. 11(0.74,1.66)	1.56(0.68,3.55), *p*=.295	0.90(0.58,1.42)	
Small for Gestational Age (SGA, <10th centile)	**1.48(1.04,2.09)**	**3.04(1.44,6.42), *p*=.003**	1.21(0.81,1.81)	**3.47(1.57,7.66), *p*=.002**
Large for Gestational Age (LGA, >90th centile	0.76(0.56,1.02)	1.04(0.57,1.89), *p*=.909	0.72(0.51,1.00)	
Gestational Age at birth (<37th week)	0.95(0.71,1.27)	0.78(0.43,1.41), *p*=.414	0.90(0.64,1.25)	
Mode of delivery (cesarean)	**0.74(0.60,0.90)**	1.10(0.74,1.63), *p*=.643	1.16(0.92,1.46)	
		**Linear Regression**		
	Unadjusted coeff (95%CI)	Interaction with gender in crude model	Adjusted coeff (95%CI)	Interaction with gender in adjusted model
Length for age Z- score	**−0.12 (−0.22,−0.01)**	−0.04(−0.24, 0.17), *p*=.723	−0.06(−0.18,0.05)	
Length centile	−**3.37(−6.21, −0.53)**	−0.36(−6.04,5.32), *p*=.901	−1.61 (−4.69,1.47)	
Head circumference for age Z- score	−0.04(−0.015,0.06)	−0.099(−0.31,0.12), *p*=.400	0.01(-0.11,0.13)	
Head circumference centile	−2.11(−4.87,0.66)	−2.39(−7.91,3.12), *p*=.396	−0.39 (−3.38,2.60)	
Birth Weight for age Z- score	**−0.11(−0.21,−0.02)**	−0.18(−0.38,0.01), *p*=.65	−0.07(−0.17,0.04)	
Birth weight centile	**−3.20 (−5.93,−0.47)**	−4.47(−9.92,0.99), *p*=.109	−1.49(−4.41,1.42)	

##### 3.1.1.2 Interaction effects

We did not find any interactions between maternal antenatal depression with sociodemographic factors. We found two interactions with sex; for SGA and length (<10th centile), with an increased odds ratio for females to be born SGA if their mothers had been depressed during pregnancy (OR 3.04 [1.44,6.42]) and more likely to be in the lower 10th centile for length (OR 2.01 [1.19, 3.40]. The effects were not significant for males (SGA OR 0.77 [0.42, 1.41] and length 0.90 [0.61, 1.31]). In the adjusted models the interaction terms remained significant. There was an increased risk for females to be SGA (OR 3.47 95%CI 1.57, 7.66) but not for males (OR 0.59, 95%CI 0.31, 1.14). For length being below the 10^th^ centile, there was an increased risk for females 2.00 [1.15,3.46] but not for males (OR for males 0.74 [0.49, 1.13].

#### 3.1.2 Severe depression

##### 3.1.2.3 Main effects

We repeated the analysis to explore progressively higher levels of depression severity and present results for women scoring between 13–16 and women scoring ≥17 on the EPDS compared to women scoring <13 on the EPDS (reference group), Table 5. The analysis revealed that for neonates of women scoring 13–16 there was no increased risk for adverse birth outcomes; and increased likelihood to deliver with caeseran compared to vaginally (OR 0.74 [0.60,0.90], Table 5). Children of women scoring 17 and above were at increased risk for a number of outcomes. We found an increased risk for neonates of women scoring 17 or above to have a lower APGAR score (OR 1.71 [1.12, 2.61], to be below the 10^th^ centile in length (OR 1.48 [1.03,2.14]), and below the 10^th^ centile in head circumference (OR 1.77 [1.17,2.93]), have lower weight at birth (standardised score, −0.19 [−0.33,−0.04], centile −5.86 [−9.91,−1.81] and to be SGA (lower 10^th^ centile, 2.12 [1.36, 3.33]) and less likely to deliver vaginally (OR 0.99 [0.64,1.51]]). Adding confounders to the models attenuated the associations between antenatal depression and child birth outcomes with the exception of increased risk of lower APGAR scores if mothers scored above ≥17 on the EPDS (OR 1.63 [1.02,2.60]), and being SGA (1.77 [1.06,2.97], Table 5.

**Table 5:** Crude and adjusted analysis for the association between maternal antenatal depression and birth outcomes by depression severity (moderate/severe) in the Pelotas, 2015 Birth Cohort.

	Logistic regression Crude OR (95%CI)				Logistic regression Adjusted OR (95%CI)			
Variables	No depression EPDS<13 *N* = 2550, 83.72%	Moderate Depression EPDS 13–16 *N* = 292, 9.59%	Severe Depression EPDS≥17 *N* = 204, 6.7%	Interaction term for gender (at moderate and severe levels of depression for females)	No depression EPDS<13 *N* = 2550, 83.72%	Moderate Depression EPDS 13–16 *N* = 292, 9.59%	Severe Depression EPDS≥17 *N* = 204, 6.7%	Interaction term for gender (at moderate and severe levels of depression for females) following inclusion of confounders
APGAR 1 min (<7)	Reference group	0.95(0.61,1.48)	**1.71(1.12,2.61)**	2.30((0.93,5.69), *p*=.071	Reference group	0.88 (0.55,1.41)	**1.63(1.02,2.60)**	
				1.25(0.53,2.9), *p*=.610				
APGAR 5 min (<7)		0.60(0.14,2.52)	0.43(0.06,3.17)	2.26(0.13,40.65), *p*=.581		0.62(0.14,2.78)	0.31(0.04,2.51)	
				1.00				
Newborn health problems (yes)		1.04(0.69,1.56)	1.20(0.76,1.89)	1.10(0.48,2.48), *p*=.827		0.87(0.56,1.36)	0.83(0.53,1.51)	
				0.75(0.29,1.94), *p*=.558				
Admitted to NICU^1^		1.10(0.70,1.73)	1.48(0.92,2.38)	0.91(0.36,2.29), *p*=.844		1.02(0.62,1.67)	1.21(0.70,2.11)	
				0.95(0.36,2.52), *p*=.916				
Length (<10th centile)		1.13(0.81,1.56)	**1.48(1.03,2.14)**	1.44(0.73,2.82), *p*=.291		0.96 (0.66,1.38)	1.19(0.78,1.79)	1.32(0.65,2.67), *p*=.444
				**3.11(1.46,6.64), *p*=.003**				**3.42(1.53,7.63), *p*=.003**
Head circumference (<10th centile)		0.67(0.36,1.25)	**1.77(1.17,2.93)**	1.18(0.33,4.13), *p*=.801		0.58(0.30,1.11)	1.42(0.81,2.50)	
				1.94(0.69,5.45), *p*=.210				
Small for Gestational Age (SGA, <10th centile)		1.05(0.64,1.72)	**2.12(1.36,3.33)**	**3.37(1.12, 10.08), *p*=.030**		0.88(0.52,1.50)	**1.77(1.06,2.97)**	**3.44(1.11,10.66), *p*=.032**
				**2.98(1.15,7.68), *p*=.024**				**3.70(1.33,10.35), *p*=.012**
Large for Gestational Age (LGA, >90th centile		0.75(0.52,1.10)	0.76(0.48,1.18)	**2.18(1.00,4.76), *p*=.051**		0.75(0.50,1.13)	0.67(0.41,1.10	2.05(0.92, 4.60), *p*=.810
				**0.31(0.11,0.90), *p*=.031**				**0.25(0.08,0.81), *p*=.021**
Gestational Age at birth (<37th week)		0.92(0.63,1.33)	0.99(0.64, 1.51)	0.72(0.34,1.53), *p*=.387		0.85(0.56,1.27)	0.98(0.61,1.57)	
				0.88(0.37,2.09), *p*=.777				
Mode of delivery (cesarean)		**0.76(0.59,0.97)**	**0.71(0.53,0.95)**	1.41(0.85, 2.32), *p*=.181		1.12(0.84,1.48)	1.22(0.87,1.70)	
				0.77(0.43,1.39), *p*=.385				
		**Linear Regression**				**Linear Regression**		
		Unadjusted coeff (95%CI)				Adjusted coeff (95%CI)		
Length for age Z- score		−0.11(−0.25,0.02)	−0.12(−0.28, 0.03)	0.08(−0.18,0.34), *p*=.543		−0.06(−0.19,0.08)	−0.07(−0.24,0.09)	
				−0.21(−0.52,0.10), *p*=.184				
Length centile		−3.11(−6.68,0.46)	−3.74(−7.96,0.47)	2.84(−4.30,9.97), *p*=.436		−1.16(−4.88,2.56)	−2.31(−6.76,2.15)	
				−5.02(−13.46,3.43), *p*=.244				
Head circumference for age Z- score		−0.01(−0.14,0.013)	−0.10(−0.26,0.06)	0.06(−0.21,0.33), *p*=.672		0.05(−0.09,0.19)	−0.04(−0.21,0.12)	0.07(−0.20,0.34), *p*=.615
				−0.31(−0.63,0.00), *p*=.054				**−0.32(−0.64,0.00), *p*=.49**
Head circumference centile		−1.10(−4.58,2.37)	−3.55 (−7.65, 0.55)	−0.04(−6.97,6.89), *p* = 991		0.54(−3.07,4.15)	−1.81(−6.13,2.50)	
				−5.91(−14.12,2.29), *p*=.158				
Birth Weight for age Z- score		−0.06(−0.18,0.06)	**−0.19(−0.33,−0.04)**	−0.03(−0.27,0.22), *p*=.833		−0.01(−0.14,0.11)	0.15(−0.31,0.00)	−0.03(−0.27,0.21), *p*=.815
				**−0.42(−0.71, −0.13), *p*=.005**				**−0.44(−0.73,−0.16), *p*=.002**
Birth weight centile		−1.35(−4.78,2.08)	**−5.86(−9.91,−1.81)**	−0.92(−7.78,5.94), *p*=.792		0.48(−3.04,4.00)	**−4.54(−8.76,−0.32)**	−0.88(−7.63,5.87**)**, *p*=.798
				**−9.87(−17.98,−1.76), *p*=.017**				**−10.66(−18.65,−2.67), *p*=.009**

1 NICU: Neonatal Intensive Care.

##### 3.1.2.4 Interaction effects

We did not find interactions with sociodemographic factors. We report interactions of child sex by depression grouping for birth weight (standardised and centile scores), being SGA, LGA and being in the lower 10^th^ centile for length. These interactions revealed an increased risk for females compared to males. Females whose mothers had severe depression (≥17) were consistently at increased risk for these outcomes. More specifically, females whose mothers had severe depression were at increased risk of lower birth weight (standardised, −0.42 [−0.71, −0.13) and centile scores (−9.87 [−17.98,−1.76], for being SGA (OR 2.98 [1.15,7.68] and for being in the lower 10^th^ centile for length (OR 3.11 [1.46, 6.64], LGA (OR 0.31 [0.11, 0.90]). For males whose mothers scored ≥17 on the EPDS: lower birth weight (standardised, 0.01 [−0.19,0.20] and centile scores −1.26 [−6.82,4.30]), for being SGA (OR 1.16 [0.55,2.46], for being in the lower 10^th^ centile for length (0.82 [0.46,1.47]) and LGA (OR 1.12 [0.66, 1.90]).

Following the addition of confounders we included the interaction terms depression X sex for the outcomes outlined above. Similarly to the unadjusted estimates, further analysis indicated an increased risk for females but not for males. Females whose mothers scored ≥17 on the EPDS were at increased risk for having lower standardised weight scores (−0.44 [−0.73,−0.16], weight centiles −10.66 [−18.65,−2.97]), and SGA (OR 3.70 [1.33,10.35]), to be in lower 10^th^ centile for length (OR 3.42 [1.53,7.63]) and LGA (OR 0.25 [0.08, 0.81]), Supplementary Table 2. We additionally found females whose mothers had experienced moderate depression to be at increased risk for being in the lower 10^th^ centile for length (OR 1.32 [0.65,2.67]) and for being SGA (OR 3.44 [1.11,10.66]). For males whose mothers scored ≥17 on the EPDS: lower birth weight (standardised, 0.05 [−0.15,0.25]) and weight centile scores (0.45 [−5.18,6.08]), for being SGA (OR 0.85 [0.37,1.98]), for being in the lower 10^th^ centile for length (OR 0.62 [0.33,1.18]) and LGA (OR 1.05 [0.59, 1.87]).

The addition of confounders led to only small decreases in the strength of the estimates between crude and adjusted analysis.

## 4 Discussion

### 4.1 Main findings

In this study, we examined the association between maternal antenatal depression and child birth outcomes in a large prospective cohort in Brazil. We examined both main effects and explored interactions with sociodemographic characteristics and child's sex. We did not find any main effects in the association of the relationship between antenatal depression and child outcomes. We did find an interaction between maternal depression and child sex; with females at increased risk for being SGA if their mothers had scored above threshold during pregnancy. Unlike previous reports we did not find an association with PTB or LBW.

We also explored this association using a higher cut-off on the EPDS to investigate higher thresholds of depression severity based on evidence that severity is associated with higher risks for child outcomes both in the antenatal and postnatal periods (Jarde et al., 2016; Netsi et al., 2018). We did find evidence of increased risk with more severe depression but overall these associations attenuated after the inclusion of confounders. Following the addition of confounders in the model we only found an increased risk of lower APGAR scores (<7) and being SGA. Depression scores between 13–16 did not raise the risk for (negative) child outcomes when examining main effects, but raised the risk when examining interactions for being in the lower 10^th^ centile for length and for being SGA for females only. An increased risk was consistently evident for children of women scoring 17 and above on the EPDS. For female newborns whose mothers experienced more severe depression during pregnancy, we found an increased risk of having lower weight scores, being SGA and in the lower 10^th^ centile for length. We did not find any evidence that sociodemographic factors moderated the association between antenatal depression and birth outcomes.

### 4.2 Interpretation

Our findings suggest that female infants exposed to severe depression during pregnancy are at increased risk of being SGA in the lower 10^th^ centile for length and for having lower weight scores. Given the evidence, that interventions in LMIC which increase birth weight and linear growth are likely to result in substantial gains in height and schooling particularly when they occur in the first 2 years of life, early identification is particularly important (Adair et al., 2013; Horta et al., 2017). Our findings highlight such a group that can be identified before birth, through screening for depressive symptoms during pregnancy, and could be prioritised for interventions early in life.

There are a number of possible explanations for our findings. First, Brazil is one the countries with the most rapid reductions in neonatal mortality (Lawn et al., 2014) and has undergone substantial changes in perinatal services in the last decades, leading to a high standard of perinatal service and improved health for all mothers. This is also reflected in some of the outcomes where we found a low variability in scores (e.g. for APGAR; at 5 min neonates in this cohort scored a mean 9.5 (standard deviation 0.8) indicating excellent health).

A recent meta-analysis reported a trend for exposure to more severe depression to be associated with higher risks for PTB and LBW (Jarde et al., 2016). The nature of the depression may be important as more severe depression may have stronger effects on foetal programming but may also place women at higher risk for postnatal depression which has been shown to impact on child development via disruptions in parenting (Stein et al., 2012). More specifically, depression that is both persistent and severe seems to substantially raise the risk of offspring adverse outcomes both in early childhood and late adolescence (Netsi et al., 2018).

A number of studies have now examined potential sex specific effects in the association between antenatal depression and child outcomes and the evidence suggests there may be different programming effects for males and females. Our finding that girls are more susceptible to the effects of antenatal depression symptoms is in line with animal studies suggesting increased reactivity of the HPA axis and cardiovascular reactivity in female offspring as well as human studies suggesting increased depressive/anxious symptoms and increased HPA activity. One potential mechanism relates to increased glucocorticoids altering the development of the foetal HPA axis, and there is evidence that there may be sex specific effects from recent research where increased maternal cortisol during pregnancy was associated with higher levels of negative emotionality and lower birth weight in girls (Braithwaite et al., 2017). These effects may have long term consequences; analysis from the 2004 Pelotas birth cohort indicated birth outcomes including being SGA, smaller head circumference and lower ponderal index to be associated with attention difficulties at age four for females only (Murray et al., 2015). Furthermore a recent analysis of a large UK–based cohort, reported depression during pregnancy to be associated with increased risks of offspring depression at 18 for females only (Quarini et al., 2016), suggesting these sex dependant differences at birth may affect a number of important developmental domains. It is important to note though that in this literature, sex specific effects have not consistently identified females at being of increased risk with some reports highlighting effects on males but not females (Li et al., 2010) and some reporting no clear sex differences (O'Connor et al., 2002).

We found a high prevalence of depression in this sample (16%) and previous analysis of this data indicates similar patterns of association with known risk factors previously reported in the literature (Coll et al., 2017). However, we found a low prevalence of suicidal ideation in this community sample (1% indicated the thought of harming themselves had occurred to them quite often, or sometimes (2.4%)). Higher levels of suicidal ideation could indicate clinically more severe depression and other community samples in LMIC indicate higher level of suicidal ideation (van Heyningen et al., 2016). Future work should investigate whether this is an important consideration in relation to birth outcomes.

Our analysis included a number of known confounders for the association between antenatal depression and birth outcomes. The attenuation of association between antenatal depression and birth outcomes following the addition of these confounders further highlights that depression is likely to occur within a constellation of other risk factors. This study specifically addressed the association between antenatal depression and birth outcomes but further in-depth investigation of the relative contribution of these confounding factors and the potential underlying mechanisms through which they may confer risk requires further investigation. Most of the confounding variables included in this analysis are routinely collected at point of care and could be used in addition to screening measures for depression to identify women and infants at risk. This also highlights the need for integrated approaches to perinatal care including perinatal mental health which address the constellation of risk factors that may be present.

### 4.3 Strengths and limitations

This is one of the few studies examining the association between antenatal depression and child birth outcomes in LMIC and one of only a handful of studies which attempts to disentangle the effects of mild or moderate depression compared to more severe levels of depression (Campbell et al., 1995; Netsi et al., 2018; Wickramaratne et al., 2011). Future studies should further investigate the role of the severity of the depression in relation to other important outcomes known to be associated with antenatal depression such as, but not limited to, brain development (Posner et al., 2016), emotional and behavioural outcomes. Furthermore research needs to address the mechanisms which underpin the association beweeen antenatal depression and birth outcomes, particularly in LMIC where antenatal depression is more prevalent as are many risk factors known to be associated with it (Herba et al., 2016). This study has a number of strengths. We utilised a large community sample of women and their children using the Intergrowth 21^st^ standards to calculate standardised and centile scores. The standards take into account sex and gestational age and are drawn from an international sample of both HIC and LMIC populations of women characterised as being of low obstetric risk. Data on depression was collected prospectively using a well-established screening measure for depression which has been validated in this population. Overall studies have not adequately considered the potential confounding role of pregnancy complications or being of high obstetric risk in the association between antenatal depression and child birth outcomes (Bindt et al., 2013; Ferri et al., 2007; Rahman et al., 2007). In this sample, we include data on gestational hypertension, diabetes and eclampsia as indicators of obstetric risk.

The study has a number of limitations. Information on whether the delivery was initiated by the healthcare provider was not available and this is potentially relevant because it may have affected our variable of gestational age. Due to the high rate of caesarean sections in this sample specifically, delivery initiated by the health care provider is very likely without necessarily indicating the foetus may have been at risk or distress. Additionally, information was not available on the overall care participants received during pregnancy such as the frequency of visits or quality of care; we are therefore not able to examine whether there were differences amongst participants in this variable and whether this was associated with birth outcomes. An important limitation of this cohort is that information on antenatal depression was available only during the second trimester. Additional points of measurement in pregnancy would have allowed us to potentially disentangle timing effects as well as whether some of the increased risks seen for infants of women with more severe symptoms of depression was related only to severity of depression or also potentially chronicity of depression.

## 5 Conclusion

In this large prospective cohort, we find evidence of an elevated risk for being SGA but did not find evidence of increased risk of other variables reported elsewhere such as PTB or length at birth for children of women experiencing depression during pregnancy. We found important sex differences primarily for children of women with more severe depression adding to the literature that females may be more susceptible to the effects of antenatal depression and stress. Maternal antenatal depression remains a significant public health concern and should be addressed not only because it is distressing to the individual and their family but it also raises the risk for future episodes of depression which are known to affect children's development, through a different set of mechanisms to antenatal depression, including parenting and the mother-child relationship.

## Role of the funder/sponsor

The funders had no role in the design and conduct of the study; collection, management, analysis, and interpretation of the data; preparation, review, or approval of the manuscript; and decision to submit the manuscript for publication.

## Author statement

Dr Netsi had full access to all of the data in the study and takes joint responsibility with Dr Coll for the integrity of the data and the accuracy of the data analysis. Dr Netsi contributed to the study concept and design, the acquisition, analysis and interpretation of data, drafting of the manuscript, critically revised the manuscript for important intellectual content, conducted the statistical analysis.

Dr Coll had full access to all of the data in the study and takes joint responsibility with Dr Netsi for the integrity of the data and the accuracy of the data analysis. Dr Coll contributed to the study concept and design, the acquisition, analysis and interpretation of data, drafting of the manuscript, critically revised the manuscript for important intellectual content, conducted the statistical analysis.

Professor Stein contributed to the study concept and design, the acquisition, analysis and interpretation of data, drafting of the manuscript and critically revised the manuscript for important intellectual content.

Dr Silveira contributed to the study concept and design, the acquisition, analysis and interpretation of data, drafting of the manuscript and critically revised the manuscript for important intellectual content.

Dr Bertoldi contributed to the study concept and design, the acquisition, analysis and interpretation of data, drafting of the manuscript and critically revised the manuscript for important intellectual content.

Dr Bassani contributed to the study concept and design, the acquisition, analysis and interpretation of data, drafting of the manuscript and critically revised the manuscript for important intellectual content.

Dr Wehrmeister contributed to the study concept and design, the acquisition, analysis and interpretation of data, drafting of the manuscript and critically revised the manuscript for important intellectual content.

Dr Domingues contributed to the study concept and design, the acquisition, analysis and interpretation of data, drafting of the manuscript and critically revised the manuscript for important intellectual content.

All authors contributed to and approved the final manuscript.

## Declaration of Competing Interest

The authors declare no conflict of interest.

## Appendix Supplementary materials

Image, application 1

Image, application 2
