# Supplementary material for: Female infants are more susceptible to the effects of maternal antenatal depression; findings from the Pelotas (Brazil) Birth Cohort Study
Source: J Affect Disord. 2020 Apr 15;267:315–24. doi: 10.1016/j.jad.2020.02.025 (PMC7103758; doi:10.1016/j.jad.2020.02.025)
Supplement: Supplementary file 2 [file mmc2.docx]

Supplementary Table 1: Characteristics of participants with depression data available and those without (for singleton pregnancies). Pelotas, 2015 Birth Cohort.

|  | EPDS data available  (n=3,046) | |  | No EPDS data  (n=1,118) | |  | Total |
| --- | --- | --- | --- | --- | --- | --- | --- |
|  |  | |  |  | |  |  |
|  | N | *%(95%CI)* |  | *N* | *%(95%CI)* |  |  |
| Mothers’ Age (years) | *n=3045* | |  | *n=1118* |  | .001 | *n=*4163 |
| <20 | 406 | 13.3(12.2,14.6) |  | 208 | 18.6(16.4,21.0) |  | 614 |
| 20-34 | 2,183 | 71.7(70.1,73.3) |  | 762 | 68.2(65.4,70.8) |  | 2945 |
| ≥ 35 | 456 | 15.0(13.8,16.3) |  | 148 | 13.2(11.4,15.4) |  | 604 |
| Living With Partner | *n* =3045 |  |  | *n=1118* |  | <.001 | *n=4163* |
| No | 392 | 12.9(11.7,14.1) |  | 199 | 17.8(15.7,20.2) |  | 591 |
| Yes | 2653 | 87.1(85.9,88.3) |  | 919 | 82.2(85.9,88.3) |  | 3572 |
| Maternal Skin Colour | *n=3042* |  |  | *n=1118* |  | <.001 | *n=4163* |
| White | 2233 | 73.3(71.7,74.9) |  | 754 | 67.4(64.6,70.1) |  | 2987 |
| Other | 812 | 26.7(25.1,28.3) |  | 364 | 32.6(29.9,35.4) |  | 1176 |
| Was this a planned pregnancy? | *n=3045* |  |  | *n=1118* |  |  | *n*=4163 |
| Planned | 1538 | 50.5(48.7,52.3) |  | 449 | 40.2(37.3,43.1) | <.001 | 1987 |
| Unplanned | 1507 | 49.5(47.7,51.3) |  | 669 | 59.8(56.9, 62.7) |  | 2176 |
| Smoking during pregnancy | *n =3044* |  |  | *n=1117* |  | <.001 | *n=*4161 |
| No | 2613 | 85.8(84.6,87.0) |  | 858 | 76.8(74.2,79.2) |  | 3471 |
| Yes | 431 | 14.2(13.0,15.4) |  | 259 | 23.2(20.8,25.8) |  | 690 |
| Alcohol consumption | *n =3043* |  |  | *n* =*1118* |  | .019 | *n=*4161 |
| No | 2832 | 93.1(92.1,93.9) |  | 1016 | 90.9(89.0,92.4) |  | 3848 |
| Yes | 211 | 6.9(6.1,7.9) |  | 102 | 9.1(7.6,11.0) |  | 313 |
| Sex of the child | *n =3043* |  |  | *n* =*1114* |  | .744 | *n=*4157 |
| Male | 1541 | 50.6(48.9,52.4) |  | 570 | 51.2(48.2,54.1) |  | 2111 |
| Female | 1502 | 49.4(47.6,51.1) |  | 544 | 49.4(47.6,51.1) |  | 2046 |
| Maternal Schooling (years) | *n =3044* |  |  | *n* =*1118* |  |  | *n=*4163 |
| 0-4 | 223 | 7.3(6.4,8.3) |  | 159 | 14.2(12.3,16.4) | <.001 | 382 |
| 5-9 | 708 | 23.3(21.8,24.8) |  | 365 | 32.6(30.0,35.5) |  | 1073 |
| 9-11 | 1112 | 36.6(34.9,38.3) |  | 313 | 28.0(25.4,30.7) |  | 1426 |
| ≥12 | 1001 | 32.9(31.2,34.6) |  | 281 | 25.1(22.7,27.8) |  | 1282 |
| Household Income | *n =3044* |  |  | *n* =*1118* |  |  | *n=4162* |
| 1 | 530 | 17.4(16.41,18.8) |  | 301 | 26.9(24.4,29.6) | <.001 | 831 |
| 2 | 617 | 20.3(18.9,21.7) |  | 216 | 19.3(17.1,21.7) |  | 833 |
| 3 | 623 | 20.5(19.1,21.9) |  | 212 | 19.0(16.8,21.4) |  | 835 |
| 4 | 635 | 20.9(19.5,22.3) |  | 206 | 18.4(16.3,20.8) |  | 841 |
| 5 | 639 | 21.0(19.6,22.5) |  | 183 | 16.4(14.3,18.7) |  | 822 |
| Parity | *n =3044* |  |  | *n =1118* |  |  | *n=*4162 |
| 1 (primiparae) | 1571 | 51.6(49.8,53.4) |  | 509 | 45.5(42.6,48.5) | <.001 | 2080 |
| 2 | 962 | 31.6(30.0,33.3) |  | 329 | 29.4(26.8,32.2) |  | 1291 |
| 3 or more | 511 | 16.8(15.5,18.2) |  | 280 | 25.0(22.6,27.7) |  | 792 |

|  | Male Newborns | | | Female Newborns | | | |
| --- | --- | --- | --- | --- | --- | --- | --- |
|  | EPDS<13  N=1285(83.4%) | EPDS 13-16  N=148 (9.6%) | EPDS>=17  N=108 (7.1%) | EPDS<13  N=1262(84%) | EPDS 13-16  N=144 (9.6%) | EPDS>=17  N=96 (6.4%) | |
| Variables |  | Adjusted Coeff (95%CI) | Adjusted Coeff (95%CI) | Adjusted Coeff (95%CI) | Adjusted Coeff (95%CI) | Adjusted Coeff (95%CI) | |
| Length (<10th centile^1^) | Reference | 0.84 (0.51,1.39) | 0.62(0.33,1.18) | **0.77(0.61,0.97)** | **1.32(0.65,2.67)** | **3.42(1.53,7.63)** | |
| Birth Weight for age Z- score^2^ |  | 0.00(-0.17,0.17) | 0.05(-0.15,0.25) | 0.00(-0.07,0.08) | -0.03(-0.27,0.21) | **-0.44(-0.73,-0.16)** | |
| Birth weight centile^2^ |  | 0.91(-3.93,5.75) | 0.45(-5.18,6.08) | -0.01(-2.15,2.14) | -0.88(-7.63,5.87) | **-10.66(-18.65,-2.67)** | |
| Small for Gestational Age* (SGA, <10th centile^1^) |  | 0.38(0.17,1.10) | 0.85(0.37,1.98) | 0.90(0.64,1.25) | **3.44(1.11,10.66)** | **3.70(1.33,10.35)** | |
| Large for Gestational Age (LGA, >90th centile^1^) |  | -0.51(0.27,0.95) | 1.05(0.59, 1.87) | 0.94(0.75,1.18) | 2.05(0.92,4.60) | **0.25 (0.08,0.81)** | |
| ^1^ Estimates are odds ratios from logistic regression  ^2^ Measure is continuous, estimates are coefficients from linear regression | | | | | | |  |

**Supplementary Table 2: Adjusted analysis for birth outcomes by sex (male/female) and by depression (moderate/severe) for outcomes with a significant depression x child sex interaction**
